# Evaluation of Organofunctionalized Polydimethylsiloxane Films for the Extraction of Furanic Compounds

**DOI:** 10.3390/polym15132851

**Published:** 2023-06-28

**Authors:** Yamile Pérez-Padilla, Manuel Aguilar-Vega, Erbin Guillermo Uc-Cayetano, Adriana Esparza-Ruiz, Marcial Alfredo Yam-Cervantes, David Muñoz-Rodríguez

**Affiliations:** 1 Facultad de Ingeniería Química, Universidad Autónoma de Yucatán, Periférico Norte Km 33.5, Tablaje Catastral 1361, Chuburná de Hidalgo Inn, Mérida C.P. 97203, Yucatán, Mexico; 2 Unidad de Materiales, Centro de Investigación Científica de Yucatán, A.C., Calle 43 No. 130 Por 32 y 34, Chuburná de Hidalgo, Mérida C.P. 97205, Yucatán, Mexico; 3 Departamento de Proyectos en Ingeniería, Universidad Internacional Iberoamericana, Calle 15, No. 36, Entre 10 y 12, IMI III, Campeche C.P. 24040, Campeche, Mexico

**Keywords:** polydimethylsiloxane, hybrid polymer, sorbent, furanic compound, characterization

## Abstract

Hybrid membranes with three different thicknesses, PMDS_C1, PMDS_C2, and PMDS_C3 (0.21 ± 0.03 mm, 0.31 ± 0.05 mm, and 0.48 ± 0.07 mm), were synthesized by the sol–gel method using polydimethylsiloxane, hydroxy-terminated, and cyanopropyltriethoxysilane. The presence of cyano, methyl, and silicon-methyl groups was confirmed by FTIR analysis. Contact angle analysis revealed the membranes’ hydrophilic nature. Solvent resistance tests conducted under vortex and ultrasonic treatments (45 and 60 min) demonstrated a preference order of acetonitrile > methanol > water. Furthermore, the membranes exhibited stability over 48 h when exposed to different pH conditions (1, 3, 6, and 9), with negligible mass losses below 1%. The thermogravimetric analysis showed that the material was stable until 400 °C. Finally, the sorption analysis showed its capacity to detect furfural, 2-furylmethylketone, 5-methylfurfural, and 2-methyl 2-furoate. The thicker membrane was able to adsorb and slightly desorb a higher concentration of furanic compounds due to its high polarity provided by the addition of the cyano groups. The results indicated that the membranes may be suitable for sorbent materials in extracting and enriching organic compounds.

## 1. Introduction

Furanic compounds are a broad class of heterocyclic molecules containing a five-membered aromatic ring with four carbon atoms and one oxygen atom with one or more substitutes. The latter include aldehydes, ketones, esters, alcohols, acids, thiols, and sulfides, in combination with pyrazines and pyrroles. Among furan, 2–FAL and HMF have been reported to be toxic and probably carcinogenic to humans. Furthermore, furan has generated significant concern due to its widespread presence in foods, which has led to increasing efforts to keep furan and their compounds’ levels as low as possible [1,2,3,4,5,6]. Furanic compounds are formed from the catalytic dehydration of glucose or fructose to 5-hydroxymethylfurfural (5–HMF) and from xylose to furfural, which can be presented as an unwanted component in transformed oil, such as oils in alcoholic beverages such as tequila [7]. The maximum furfural limit in alcoholic beverages is 5 mg/100 mL. It is also present in heated or thermally processed foods such as coffee, canned vegetables, roasted grains, fruits, and honey, among others [8,9,10]. 

Nowadays, several separation methods, including reverse osmosis, electrodeposition, coagulation, precipitation, electrocoagulation, coagulation–flocculation–sedimentation, biosorption, and adsorption have been applied for the removal of chemical analytes; among them, the absorption method has been considered adequate and widely applied due to its low cost, and high efficiency [11,12].

In recent years, research has been focused on developing new organic–inorganic materials for use in various fields due to their properties being the result of a combination of the properties of their constituent materials. The sol–gel process is an easy widely probed method that offers modification in organic and inorganic materials, variability and applicability for hybrid material elaboration, and good resistance at high temperatures. The sol–gel method is based on yielding an inorganic or hybrid polymer from metallic and/or inorganic alkoxides through a series of simple chemical reactions at relatively low temperatures (20 to 150 °C) [13,14,15,16].

Sol–gel is a process in which a liquid solution, “sol,” is converted into a solid phase, “gel” [15]. Sol–gel technology offers efficient and high-purity formation of nanopowder, fibers, solid structures, and thin film coatings. Potential applications of sol–gel technology are found in a wide range of sectors, such as pharmaceuticals, medicine, construction, aerospace, transportation, the food industry, optics, agriculture, semiconductor devices, catalysis, and biotechnology [16]. Organic–inorganic hybrid materials have attracted considerable interest in recent years because they can enhance physicochemical, thermal, and mechanical properties. Organic functional silanes can generally improve thermal stability, hardness, mechanical properties, and adhesion to specific organics other than the sol–gel, ensuring an optimal hybridization in the process where it will be applied [15,16,17]. 

Different extraction methods for organic compounds from aqueous matrices based on polydimethylsiloxane (PDMS) or other polymeric supports have been developed, mainly aiming at reducing the solvents and handling faster and easier sample preparation procedures. One of the first extraction techniques employing PDMS is solid-phase microextraction (SPME), proposed by Belardi and Pawliszyn in 1989 [18,19]. The PDMS is a linear polymer. In the PDMS/TEOS network, the tetraethoxysilane (TEOS) generates a silica cluster, which crosslinks the PDMS linear chains, generating a three-dimensional structure. PDMS and composite membranes (PDMS/TEOS) have been used as supporting membranes in the latter case.

The hybrid membranes consist of hydrophobic PDMS chains crosslinked by polar organic clusters. Their design is an alternative approach to producing multifunctional materials with polar and non-polar nanodomains, leading to different intrinsic properties and thus to many applications [20,21]. Inorganic–organic hybrid polymers are increasingly crucial in materials science because they combine the properties of the materials that make them up. A critical challenge in designing these inorganic–organic hybrid systems is controlling the blend between the two different phases [22,23]. 

Hybrid polymers in the form of a membrane have a great variety of applications in different areas such as analytical chemistry, food, industry, and pharmaceutical industry, among others. In particular, in the extraction of analytes, several analysis techniques, both classical and instrumental, require having the sample in dissolution to separate the analytes of interest from the matrix. Sample pretreatments transform it into an appropriate form for further analysis; however, this stage requires more time in the analytical process and the consumption of considerable amounts of solvents, which increases costs and pollutes the environment [24]. Several techniques have been studied to help counteract the sample treatment problem, including methods such as solid phase extraction (SPE) and solid phase microextraction (SPME), which can adsorb and concentrate organic analytes with a minimum amount of material, seeking to reduce working times and the number of solvents at the sample treatment stage [25,26,27,28]

Previously, a no-porous hybrid-polymeric coating based on PDMS–OH and cy-anopropyltriethoxysilane (CPTEOS) or aminopropyltriethoxysilane (APTES) or triethoxyphenylsilane (TEPS) with sorbent properties was studied using the stir bar sorptive extraction (SBSE) technique. In all cases, furanic derivatives were moderately extracted from water, but when extracted from isooctane, they showed adsorption capacity in the order of CPTEOS > APTES > TEPS [29,30]. 

These functional groups were selected to increase the polarity of the sorbent phase and thus improve its sorption capacity towards polar compounds as the furanic ones. It was found that the contact area in the bars did not favor the interaction with the furanic compounds due to the fact that most functional groups were inside the bar and were not available to interact with the analytes. Thus, new techniques are needed, such as membrane sorption, which offers a larger contact area and possibly more functional groups on the surface to interact with the analytes of interest with the CPTEOS. This study aimed to evaluate the performance of a hybrid membrane synthesized from PDMS and CPTEOS as a sorbent in the analytical pretreatment for the determination of furan compounds. At the same time, the effect of membrane thickness and the evolution of their sorbent material properties was assessed. A description of the determination of the appropriate conditions for PDMS/CPTEOS hybrid membrane preparation by the sol–gel technique, as well as its characterization, was performed, followed by the studies of the sorption behavior of furan compounds. 

## 2. Materials and Methods

### 2.1. Reagents

Polydimethylsiloxane, hydroxy-terminated (PM 550, PDMS-OH), cyanopropyltriethoxysilane (CPTEOS), and dichloromethane (DCM) were all obtained from Sigma-Aldrich Co. Trifluoracetic acid (TFA), HPLC grade methanol, HPLC grade acetonitrile (ACN), and HPLC grade water, were provided by J.T. Baker. Sodium hydroxide (NaOH) from Fluka Analytical and hydrochloric acid (HCl) from Meyer were also used. All reagents were at least 98% pure.

### 2.2. Synthesis of the Hybrid Membranes

Membranes with different thicknesses were synthesized by varying the volume of sol–gel solutions CPTEOS:PDMS (3:1 molar ratio): (PDMS_C1), (PDMS_C2), and (PDMS_C3). To synthesize the hybrid polymeric membrane (PDMS_C1), we proceeded as follows: A solution of PDMS-C1 in a 50 mL Corning tube was prepared by mixing 631.5 µL of PDMS-OH and 900 µL of DCM. The mixture was stirred for 30 s in a Genie 2.0 vortex. Next, 783 µL of CPTEOS was added and stirred for 1 min. Subsequently, 150 µL of trifluoroacetic acid (TFA 95%) was added and stirred for 2 min. 

The solution was placed in an ultrasonic bath for 5 min. Then, the solution was transferred in a 6.5 cm diameter tetrafluoroethylene terephthalate mold to obtain the membrane and left to stand for ten days on a smooth leveled surface at 25 °C. During this time, a glass funnel was placed over the mold to control solvent removal and to keep impurities out. Final drying was completed in a vacuum oven using a heating protocol of 60 °C for 24 h, then increased to 120 °C for another 24 h. Finally, the hybrid membrane was left at 180 °C for 4 h. The same procedure was followed to prepare the 4 mL (PDMS_C2) and 6 mL (PDMS_C3) polymeric membrane adjusting the volume relation of the monomer, alkoxysilane agent, and solvents.

### 2.3. FTIR

Fourier transform infrared spectroscopy (FTIR) analysis was made on a Fourier transform infrared spectroscope model Thermo 380 FT-IR using the total attenuated reflectance method (ATR) on a spectral interval of 4000–650 cm^−1^ with a resolution of 4 cm^−1^, scan number 60, and scan target 32.

### 2.4. Thermogravimetric Analysis

Thermogravimetric analysis was performed to determine the thermal stability of the membrane on a Netzsch equipment model STA 449 F3 Jupiter. It was carried out in an ultra-high purity nitrogen atmosphere with a 20 mL/min flow, in a temperature range from 50 °C to 800 °C, and with a heating speed of 10 °C/min.

### 2.5. Contact Angle

The sessile drop method was used to determine the contact angle value in the hybrid membranes. Dataphysics OCA 15EC contact angle equipment was used for this measurement. The film strip was placed on a smooth and utterly horizontal base. Each measurement was performed with distilled water (5 μL) on the film. Once the drop was deposited, the contact angle was measured by making a digital capture using the equipment’s software. Ten measurements were made for each membrane and the average and standard deviation of the contact angle was reported.

### 2.6. Thickness Measurement

A Mitutoyo micrometer (H-2781) was used for the thickness measurement of the polymer membranes. The measurement was taken at 9 points distributed in the polymer membrane and the average and standard deviation of the results obtained by the equipment were reported. 

### 2.7. pH Stability

Stability tests for different pH solutions (1, 3, 6, and 9) were carried out. Hybrid membranes were cut into 0.5 cm × 3 cm and immersed in the different solutions for 3, 6, 24, and 48 h. To ensure the membrane had no moisture, they were dried in a convection oven at 80 °C for 3 h before testing. The variation of the weight of the membranes between the beginning and the end of the immersion was determined on an analytical balance (Sartorius Model 54). The tests were repeated three times. The weight variations and the standard deviations were reported.

### 2.8. Stability to Solvents

The solvents evaluated were methanol (MeOH), water (grade HPLC), and acetonitrile (ACN). The membranes were immersed in solvents and the weight variation of the membranes was determined when two different tests were carried out; the membranes were immersed into the solvents and vortex stirring at 15 and 45 min and an ultrasonic bath for 30 and 60 min, respectively. The tests were carried out in triplicate.

### 2.9. Analyte Extraction 

The extraction test was performed by depositing a polymeric membrane (1 cm × 1 cm trimmed into an Eppendorf tube with 20 µL of a polar organic compound solution: 5-(hydroxymethyl) furfural (5–HMF), furfural (2–FAL), 2-furilmethylKetone (2–FMC), 5-methyl furfural (5–MFA), methyl 2-furoate (FEMA) at a known concentration (1.0 µg mL^−1^), and 1980 µL of a 30% NaCl saline solution (*w*/*v*). The solution was shaken in a vortex for 3 h. Then, the polymer membrane was removed from the solution and placed in an Eppendorf tube with 500 µL of a methanol:water mixture 1:1. Again, the membrane was stirred in a vortex for 20 min. The membrane was removed and the solution was filtered with a 1 mL syringe. The extraction was performed by taking a 20 µL aliquot and it was injected into a high-pressure liquid chromatograph (HPLC model 1100, Agilent Technologies) with a diode array detector (G1315B), degasser (G1379A), and quaternary pump (G1311A). The specific conditions used in the chromatographic analysis are described in Table 1. 

## 3. Results

### Synthesis of PDMS_CX Hybrid Membranes by Sol–Gel Solution

**Scheme 1:**
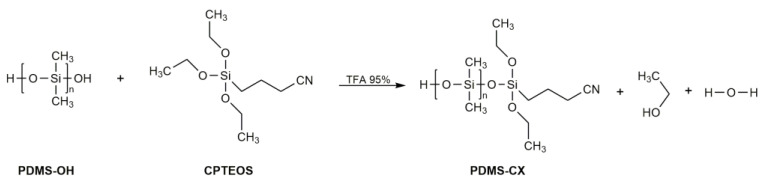
General reaction scheme for the synthesis of hybrid polymers by sol–gel.

Three hybrid membranes with different thicknesses, based on polydimethylsiloxane with terminal hydroxides (PDMS-OH) and cyanopropyltriethoxysilane (CPTEOS), were synthesized; translucent membranes, free of pores and cracks, were obtained with the different synthesized thicknesses. According to the literature, these characteristics corresponded to an adequate sol–gel reaction [14,24]. The following reaction scheme (Scheme 1) showed the sol–gel synthesis route. First, catalytic hydrolysis of CPTEOS by TFA occurred, followed by condensation via a reaction between the hydrolyzed CPTEOS and PDMS-OH to form an evolving sol–gel network. The curing conditions of the material were responsible for the homogeneous non-porous structure of the obtained membranes.

**Figure 1:**
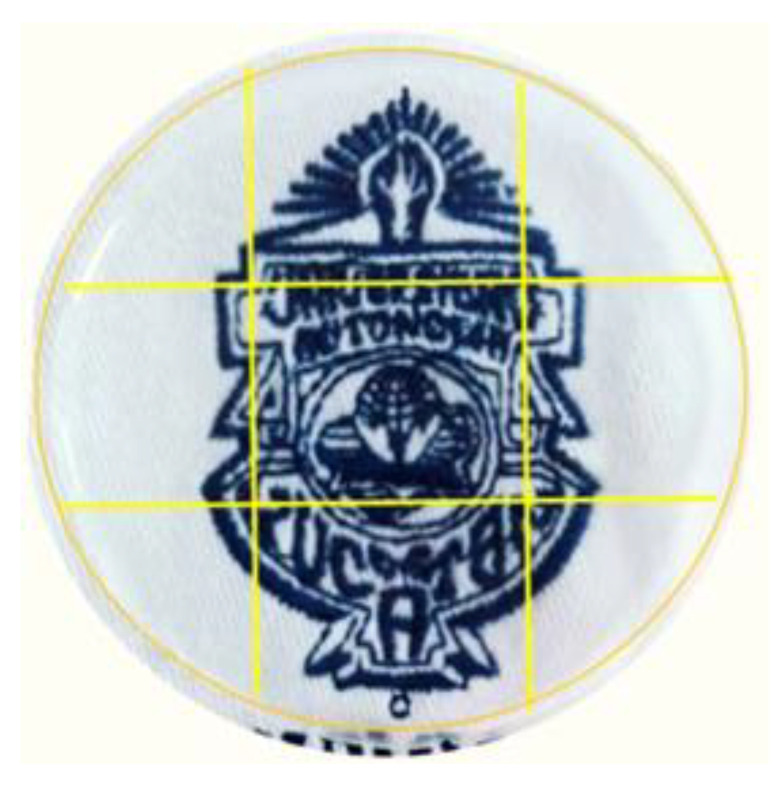
Image of synthesized membrane PDMS-C1 with thickness measurement marks.

The thickness measurement on the PDMS_CX membranes was carried out by dividing them into nine sections; an image of which can be seen in Figure 1.

**Figure 2:**
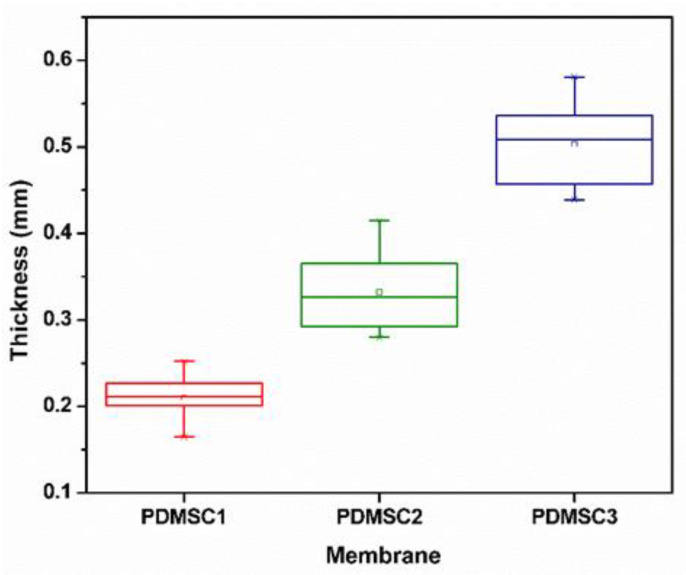
The standard deviation of the thicknesses obtained in the membranes.

Figure 2 shows membrane thickness results and the standard deviation (SD). Membrane PDMS_C1 showed 0.22 +/− 0.05 mm thickness SD. PDMS_C2 had a 0.33 +/− 0.05 mm and PDMS_C3 showed a 0.55 +/− 0.05 mm thickness. Thickness homogeneity for PDMS_CX membranes was achieved by controlling the amount of solution deposited in a properly leveled mold, followed by the elimination of solvent during the forming process. We were able to identify the synthesis conditions and could corroborate the reproducibility of the process. 

**Figure 3:**
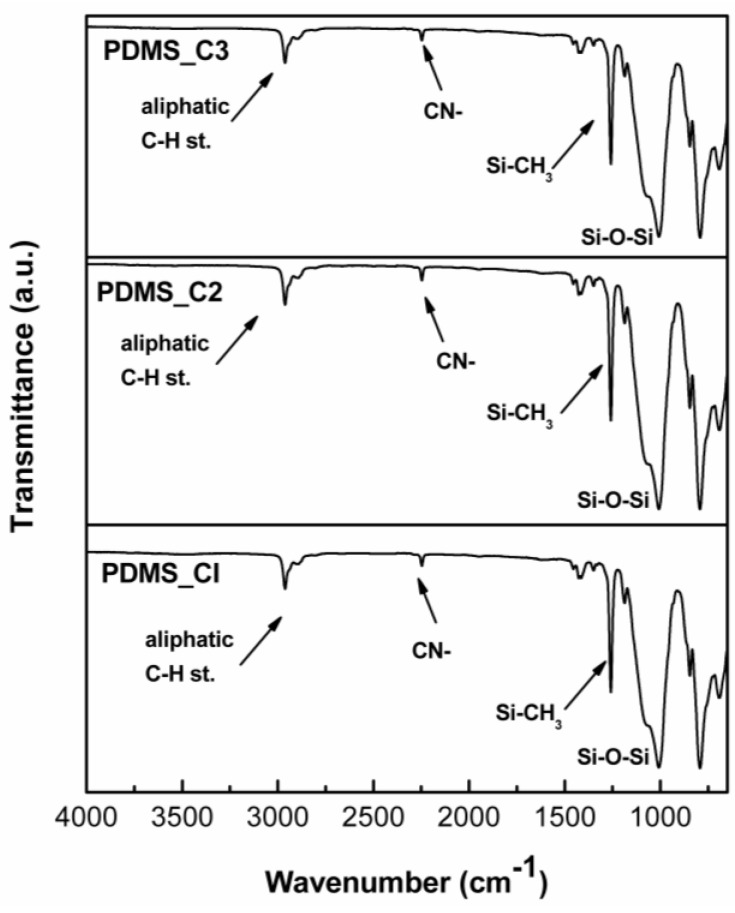
FTIR–ATR spectrums of PDMD_CX hybrid polymeric membranes.

The FTIR spectra of hybrid polymeric membranes prepared with PDMS-OH and CPTEOS are depicted in Figure 3. Based on previous studies from our research group and a comprehensive literature review, the overlapping bands observed at 1005 and 1090 cm^−1^ were attributed to the Si-O-Si groups present in both PDMS-OH and the hybrid polymer PDMS_CX. Additionally, the band at 1095 cm^−1^ signified the network structure of the synthesized hybrid polymer. The band corresponding to the stretching methyl -CH groups of PDMS were observed at 2962 cm^−1^. The band corresponding to the aliphatic Si-CH_3_ groups was located at 1261 cm^−1^. The band corresponding to the cyano group -CN was observed at 2250 cm^−1^. These results were similar to those obtained for a stir bar coating prepared with PDMS previously reported by our research group and others in the literature [29,30,31,32]. Notably, no discernible differences were observed in the spectra concerning the variation in membrane thickness.

**Figure 4:**
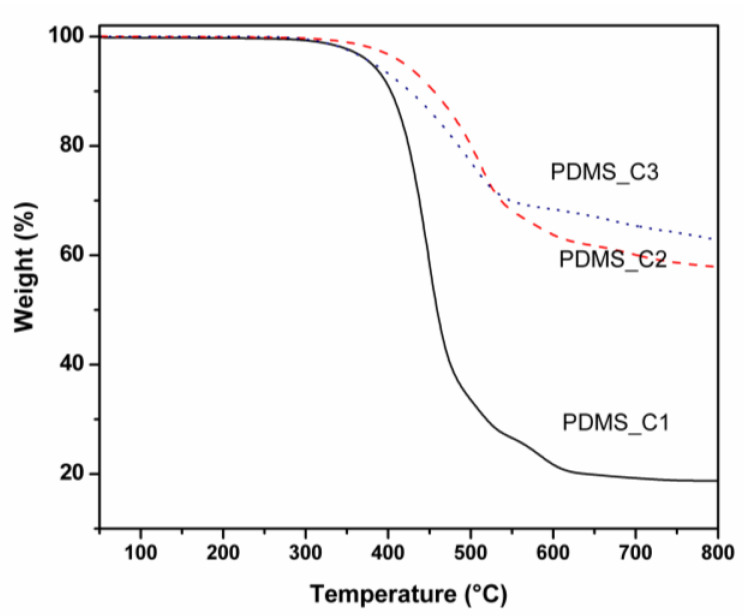
PDMS_Cx thermogram for hybrid polymeric membranes.

Figure 4 shows the thermal decomposition of the hybrid membranes with PDMS and cyanopropyltriethoxysilane. The thermal degradation curves showed a very high thermal resistance with an onset of decomposition temperature, T_d_, at 400 °C due to the decomposition of the organic material contained in the hybrid membrane. Several studies have reported that hybrid membranes showed an initial weight loss at 300 °C that was related to the decomposition of the organic hybrid membrane; these results were reported by Ballistreri et al. [33] and other studies conducted by our working group when an active coating on a stir bar was prepared with these monomers. It is essential to observe that the material did not show weight losses related to residual solvent or humidity as others have reported, indicating a good curing process.

A comparison of the thermal behavior of the different membranes is also shown in Figure 4. The membrane thickness variation did not cause differences in stability as expected. This indicated that the methodology followed for curing the hybrid membrane was adequate. The thickness variations reflected changes in the percentages of residual mass attributed to the inorganic phase, demonstrating that crosslinking was greater, as this variation increased and agreed with the number of monomers added.

Contact angle in PDMS_CX hybrid membranes was conducted to determine the synthesized polymers’ wettability and hydrophilicity. According to the literature, the contact angle value for PDMS is 100° [31]. The results in Table 2 show that adding the alkoxylate agent reduced the contact angle values when the membranes were evaluated with distilled water. 

Contact angle values for PDMS_CX hybrid membranes with different thicknesses did not show any significant difference between them. Additionally, it could be confirmed that the thickness did not influence this property. The values obtained in this study were lower than those reported by Burgos et al. [29], 74 ± 1.78°, and 77.4 ± 1.2° by Avila et al. [34], respectively, for the evaluation of a stir bar coating, using the same precursors. This fact was attributed to the cyanopropyltriethoxysilane group´s high polarity, which increased the hydrophilic capacity of the membranes and caused a contact angle to decrease value on PDMS when a hybrid polymer was formed with the alkoxylate agent.

**Figure 5:**
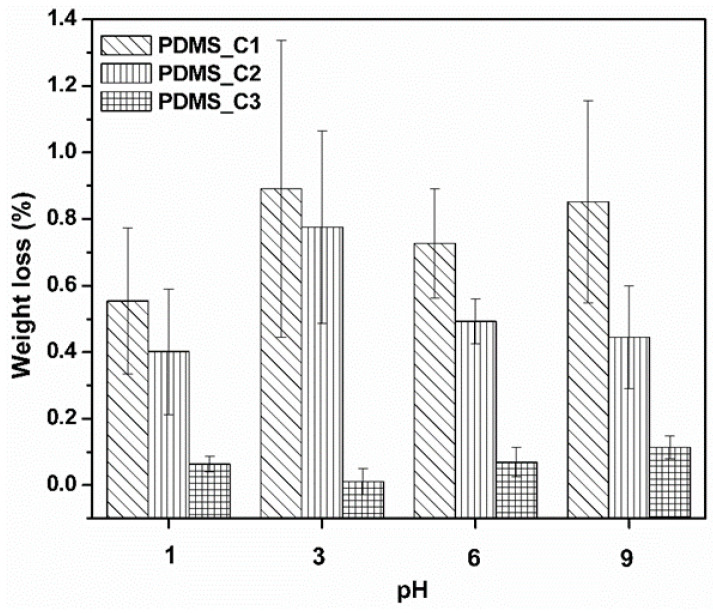
Stability of PDMS_C1, PDMS_C2, and PDMS_C3 membranes subjected to various pHs after 48 h of treatment.

The hybrid membranes were synthesized (Figure 5). PDMS_C1, PDMS_C2, and PDMS_C3 membranes presented good stability at the submitted pH (1, 3, 6, and 9) for up to 48 h of exposure with a mass loss of less than 1% *w*/*w*. It was also observed that the membrane structure was unaffected and did not suffer fractures, indicating that under these conditions, it could be evaluated without affecting the efficiency in the application corresponding to the extraction and continued to maintain its characteristics.

When the tests were carried out, we observed that PDMS_C3 membranes suffered minor damage compared with the thinner membranes. Likewise, the membranes with lower thickness, PDMS_C1 and PDMS_C2, were more vulnerable to acidic and alkaline pH effects, while the PDMS_C3 membranes were very stable regarding mass loss. 

The importance of sorbent materials lies in the stability to which they can be exposed and to the several pHs (acidic or alkaline media) during the extraction of ionizable compounds; based on this, these PDMS_C1, PDMS_C2, and PDMS_C3 membranes may be suitable as an alternative as a sorbent material for analyte extraction.

**Figure 6:**
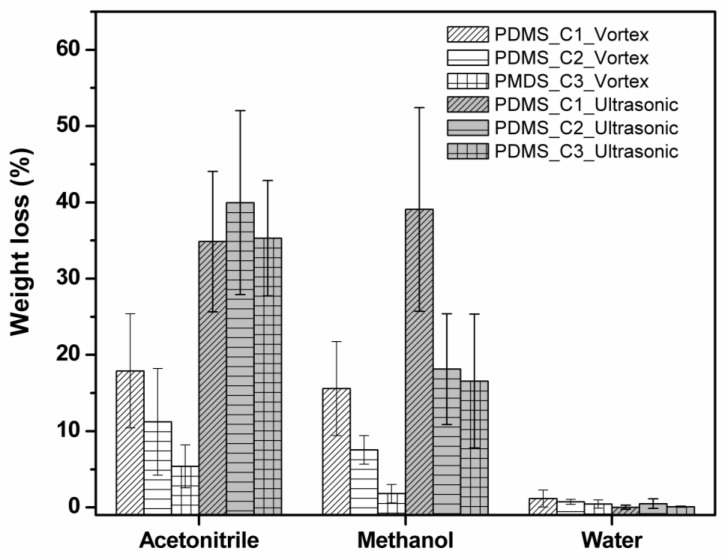
Weight loss percentage in PDMS_C1, PDMS_C2, and PDMS_C3 membranes; evaluation after immersion in different solvents and treatment: vortex 60 min; ultrasonic bath 90 min.

The results of the PDMS_CX membrane immersion in different solvents after indicated times are shown in Figure 6, as well as the immersion bath treatments in both vortex and ultrasonic stirrings. The membranes were more stable when immersed in solvents and vortexes than those subjected to ultrasonic baths for the different thicknesses tested. In the vortexes’ agitation of solutions, the movement was transmitted to the liquid, generating a vortex in the sample; this allowed vigorous mixing of the product. Stirrers aimed to force liquids or gases into motion through mechanical force. In the ultrasonic bath, an electric current transmitted its energy to a mechanical system that converted it into high-intensity vibrations that generated ultrasound waves. The ultrasound, in turn, generated vibrations in the target material. If it contained liquids, millions of microscopic bubbles would be generated, which underwent rapid expansion and collapse processes that could transmit their energy to other materials, causing erosion on their surfaces. 

In addition, the results showed that the highest losses occurred in both treatments when the membranes were immersed in acetonitrile. For PDMS_C1, a total mass loss of 17.9% was obtained, higher than PDMS_C2 with 11.22% and PDMS_C1 with 5.34% *w*/*w* of total mass loss, respectively, on vortex treatment. PDMS_C1 membrane immersed in an ultrasonic bath of acetonitrile solvent showed 49% *w*/*w* mass loss, reflected in a fracture in the polymeric material with a higher mass variation. However, according to Figure 6 and the standard deviation, the membranes of different thicknesses evaluated immersed with acetonitrile in the ultrasonic bath did not show differences in weight loss. The solubility of polymers in solvents was more complex due to the high molecular weights; the solvent molecules penetrated the polymer, sometimes swelling it and forming a gel until dissolution was achieved. 

In addition, protic solvents are generally very polar molecules that contain acidic protons (H+) and, therefore, can form hydrogen bonds with solutes. On the other hand, aprotic solvents are solvents that do not contain acidic hydrogens and, therefore, cannot form hydrogen bonds. Acetonitrile is an aprotic polar solvent; methanol and water are polar solvents. According to the results, the hybrid polymer interacted more with acetonitrile than with methanol and water. The trend of the mass variation for the solvent under test in the vortex agitation treatment and the ultrasonic bath was ACN > MeOH > H_2_O. In general, the PDMS_C1 membrane was more affected than expected for both treatments since it had a lower thickness and resistance to these solvents (Figure 5). The results suggested that when the thickness of the membrane was lower, it could suffer damage that compromised its structure in both treatments.

**Figure 7:**
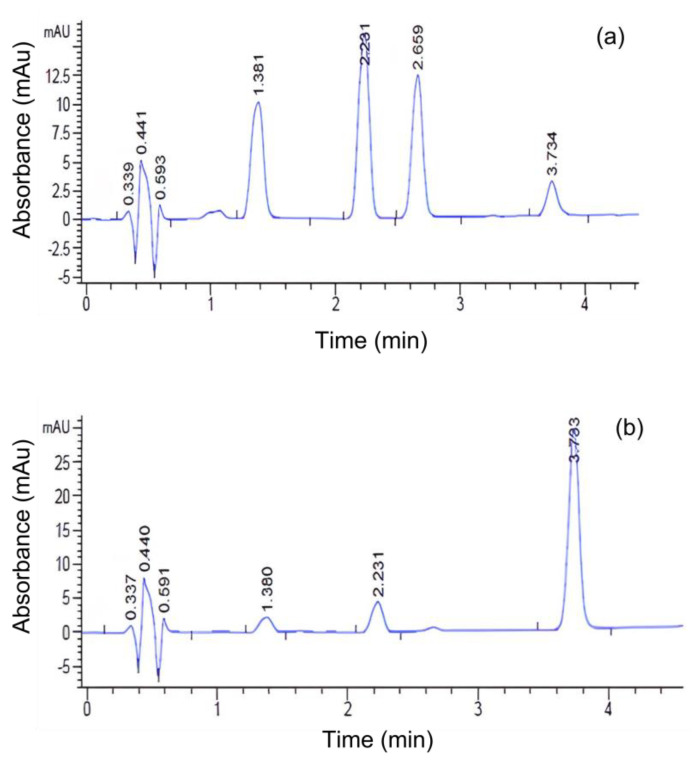
Typical chromatogram of the furanic compounds after their extraction from aqueous solution with PDMS_C3 membrane (0.48 ± 0.07 mm) (**a**) and their desorption with methanol: water (1:1), (**b**). Above, 278 nm; below, 248 nm.

Table 3 and Figure 7 present the results of the analysis of furanic compounds in the extracts after applying the membrane for the analytical pretreatment. The goal was to achieve an enrichment factor of four because the extraction step involved 2 mL of furanic (1 µg/mL) and the desorption step involved 0.5 mL of methanol: water (1:1). However, enrichment factors higher than two were obtained only for FEMA and 5–MFA, regardless of the thickness. The RSD of the extraction experiments ranged from 0.3 to 7%. 

The enrichment factors for FEMA and 5–MFA were ascribed to their high affinity toward the membrane based on PDMS. They had log *p* values of 1.0 and 0.67, respectively, and were relatively prone to interact with lipophilic materials. The dispersion forces between PDMS and cyanopropyl groups with furanic compounds could drive the affinity. In addition, the thickness was directly proportional to the concentration of FEMA and 5–MFA in the extracts. In addition, 2–FAL and 2–FMC showed the highest concentration with PDMS_C3 (0.48 ± 0.07 mm). This could be related to the increment of cyanopropyl groups as the thickness increased; as a result, there were more sites for interacting with the compounds. In contrast, 5–HMF was not detected in the final extracts because it was the most hydrophilic compound (log *p* = −0.09), regardless of the thickness. The interactions between 5–HMF and PDMS membranes were not strong enough to break the hydrogen bonding between the water molecule and the -OH group of 5–HMF. 

## 4. Conclusions

Three different hybrid membranes with different thicknesses (0.21 ± 0.03 mm, 0.31 ± 0.05 mm, and 0.48 ± 0.07 mm) were obtained by the sol–gel method using polydimethylsiloxane, hydroxy-terminated, and cyanopropyltriethoxysilane. The presence of cyano, methyl, and silicon–methyl groups was corroborated by FTIR analysis. The contact angle results on PMDS_C1, PMDS_C2, and PMDS_C3 hybrid membranes corroborated the hydrophilic character. The solvent resistance test, conducted with 45 and 60 min exposures to vortex and ultrasonic treatments, respectively, revealed a distinct preference order for solvents: ACN > MeOH > H_2_O. Additionally, when subjected to various pH conditions (1, 3, 6, and 9) over 48 h, the membranes demonstrated exceptional stability, exhibiting minimal mass losses of less than 1% *w*/*w*. These findings highlighted the robust performance of the membranes under different solvent and pH environments, further validating their suitability for practical applications. The thermogravimetric analysis showed that the material tended to lose 90% of its initial mass at 600 °C. Finally, the sorption analysis showed its capacity to adsorb furfural, 2-furylmethylketone, 5-methylfurfural, and 2-methyl 2-furoate. The thicker membrane could adsorb and slightly desorb a higher concentration of furanic compounds due to its high polarity provided by the addition of the cyano groups. These results confirmed that the synthesized membranes have the potential to be applied in the extraction of polar compounds as sorbent materials.

## Figures and Tables

**Table 1:** Chromatographic and detection conditions in the experimental section.

Column	Mobile Phase	Circumvention: Gradient	Injection Volume	Analytical Wavelength
**C18 5 µm, 4.6 mm × 50 mm**	A: Acetonitrile B: Water	0.4 min 95% B 4–5 min 80% B 4–5 min 80% B 7 min 95% B	20 µL	278 nm, 265 nm, 248 nm

**Table 2:** Contact angle average of PDMS_CX hybrid membranes.

PDMS_CX Membranes	Contact Angle (°)
PDMS_C1	68.45 ± 2.14
PDMS_C2	68.29 ± 2.44
PDMS_C3	68.04 ± 2.46

**Table 3:** Furanic compound concentration after extraction from aqueous solution with PDMS_C1, PDMS_C2, and PDMS_C3 polymeric membrane and subsequent desorption with 0.50 mL MeOH: H_2_O (1:1).

Furanic Compound	λ_Analytical_ (nm)	t_R_ (min)	Average Concentration (ppm)		
			PDMS_C1 (0.22 mm)	PDMS_C2 (0.33 mm)	PDMS_C3 (0.55 mm)
**5–HMF**	278	1.040	nd	nd	nd-
**2**–**FAL**	278	1.381	0.58 ± 0.03	0.58 ± 0.06	1.08 ± 0.02
**2–** **FMC**	278	2.231	1.01 ± 0.06	1.02 ± 0.11	1.66 ± 0.03
**5** **–MFA**	278	2.659	1.45 ± 0.06	1.51 ± 0.15	2.30 ± 0.03
**FEMA**	248	3.734	2.79 ± 0.01	3.06 ± 0.21	3.78 ± 0.05

t_R_—retention time; λ—analytical wavelength, nd—not detected.

## Data Availability

The data presented in this study are available on request from the corresponding author.
